# Characterization of a rhodopsin-phosphodiesterase from *Choanoeca flexa* to be combined with rhodopsin-cyclases for bidirectional optogenetic cGMP control

**DOI:** 10.1016/j.jbc.2025.108401

**Published:** 2025-03-11

**Authors:** Nicolas Liem, Anika Spreen, Arita Silapētere, Peter Hegemann

**Affiliations:** Institut für Biologie, Humboldt University of Berlin, Berlin, Germany

**Keywords:** rhodopsin, phosphodiesterases, optogenetics, cyclic GMP, second messenger, electrophysiology, CNG channel, photoreceptors

## Abstract

Rhodopsin phosphodiesterases (RhPDEs) were first discovered in the choanoflagellate *Salpingoeca rosetta*, but their physiological role remained unknown. Their light-dependent modulation was found to be low, limiting optogenetic application. However, recent *in vivo* studies in the choanoflagellate *Choanoeca flexa* revealed a strong linkage of RhPDE to the actomyosin-mediated contraction and colony sheet inversion and identified downstream cGMP effectors. Through screening various RhPDE variants from *C. flexa*, we identified four photomodulated PDEs of which *C*. *flexa* RhPDE1 (CfRhPDE1) revealed the highest cGMP affinity and the most pronounced light regulation with *K*_m_ values of 1.9 and 4.4 μM in light and darkness. By coexpressing CfRhPDE1 with the rhodopsin-guanylyl-cyclase from the fungus *Catenaria anguillulae* and a cyclic nucleotide-gated ion channel from olfactory neurons in ND7/23 cells, we demonstrate bidirectional dual-color modulation of cGMP levels and ion channel conductance. Together with spectroscopic characterization, our fast functional recordings suggest that the M-state of the photocycle initiates functional changes in the phosphodiesterase domain *via* rapid rhodopsin-PDE coupling. With efficient expression and 3.5 s lifetime of the active state, this protein provides high photosensitivity to the host cells. This demonstrates that RhPDEs can regulate cGMP signaling in mammalian cells on a subsecond timescale, closing a present gap in optogenetics and assisting researchers in setting up multicomponent optogenetic systems for bidirectional control of cyclic nucleotides.

In many organisms, photosensing relies on rhodopsins, which are membrane-bound retinal proteins found in many kingdoms, such as archaea, bacteria, algae, fungi, and mammals (1). The rhodopsin may act as a light-gated ion channel, a light-driven ion pump to actively or passively alter the membrane potential, or they activate cytosolic signaling proteins like G-proteins, transducers, or enzymes (1). Heterologous expression of photosensors has endowed cells with photosensitivity so that researchers can control cellular signaling with light at unprecedented temporal and spatial resolution, a technology which is called optogenetics (2). Besides naturally found photoreceptor enzymes, optogenetic tools have been created by means of chimeric proteins. Based on structure–function studies of a bacteriophytochrome and the GAF-activated phosphodiesterase HsPDE2A, their photosensory domain and catalytic phosphodiesterase (PDE) domain were combined into the light-activated phosphodiesterase, LAPD (3, 4, 5). Upon illumination with 690 nm light, its activity increases up to 6-fold, which is based on an increase in the maximum turnover number *k*_cat_. Its *K*_m_ value is large in darkness and declines slightly under illumination for both cAMP and cGMP, which renders LAPD to be unselective. Thus, LAPD is valuable for cAMP modulation but less appropriate for the control of cGMP, which is typically present in cells at a 10-100-fold lower concentration than cAMP (6).

The potency of a valuable optogenetically applicable enzyme can be characterized by several parameters: First, the resulting enzymatic reaction should be fast so that low or moderate expression levels are sufficient. This means according to classic Michaelis–Menten kinetics (7), the ratio *k*_cat_/*K*_m_ must be large, which is also called the catalytic efficiency. Second, the enzyme should be selective. Efficient regulation of cGMP by a phosphodiesterase requires a large ratio of (*k*_cat_/*K*_m_)_cGMP_/(*k*_cat_/*K*_m_)_cAMP_ called the catalytic selectivity for cGMP. An only modestly cGMP-selective phosphodiesterase would effectively hydrolyze cAMP rather than cGMP, and the experimentalist would perturb cAMP signaling instead of cGMP pathways due to the higher concentrations of cAMP in most cells. Third, the ratio of the catalyzed reaction velocities under illumination *versus* darkness should be large. Ideally, the enzyme activity in darkness (D) is negligible, and the system is only perturbed when illuminated (L). This ratio is often referred to as the fold change (fc), and for optogenetic enzymes, it is the light/dark fc, which is given by the ratio of catalytic efficiencies at steady state conditions:

fc = (*k*_cat_/*K*_m_)_L_/(*k*_cat_/*K*_m_)_D_

Although cAMP signaling has been studied by applying LAPD in combination with the soluble photo-activated cAMP-cyclase bPAC from the soil bacterium *Beggiatoa* sp. (8, 9), bidirectional cGMP signaling has not been described so far, despite the availability of light-activated guanylate cyclases and cGMP phosphodiesterases (10, 11). Moreover, robust physiological evidence supports signaling pathways that rely on a rhodopsin-guanylyl cyclase (RhGC) (12) or cGMP-specific rhodopsin phosphodiesterase (RhPDE) (Fig. 1*A*) (13, 14), suggesting similar potential for optogenetic applications. Indeed, raising cGMP levels optogenetically to open cyclic nucleotide-gated (CNG) channels with RhGCs has already been demonstrated (15). This raises the question of whether light-dependent degradation of cGMP by RhPDEs could enable bidirectional optogenetic control of CNG channel opening and closure. Here, we characterize *Choanoeca flexa* RhPDE1 (CfRhPDE1) through *in vitro* assays and complement these data with a spectral characterization of the dark state and catalytically active signaling state. These findings allowed us to set up a dual-color, three-component optogenetic system for bidirectional control of cGMP, utilizing an RhGC and a CfRhPDE1 to regulate olfactory CNG channels on a subsecond time scale.

**Figure 1:**
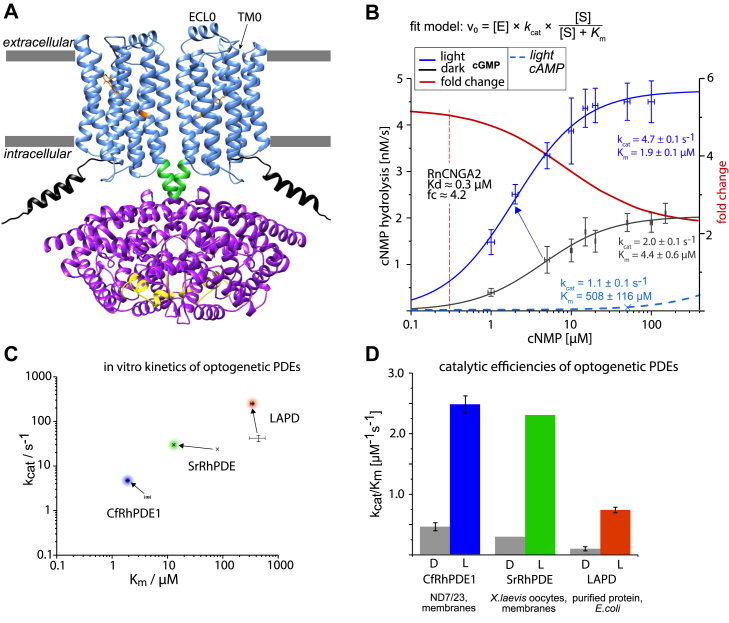
**Overall structure of CfRhPDE1 and experimental *in vitro* characteristics**. *A*, structural model generated using AlphaFold 2.0. *Black*, N-terminal region; *blue*, rhodopsin domain; *green*, linker sequence; *purple*, PDE-domain; *yellow*, H-loops of PDE domains; *orange*, all-*trans*-retinal bound to Lys276; *B*, experimental *in vitro* enzyme kinetics of CfRhPDE1 and the resulting fold change. Data were normalized to 1 nM enzyme concentration. n = 3 for each cGMP reaction tested. The *dashed line* indicates an estimate for the *K*_d_ of the C460W mutant of RnCNGA2; *C*, experimental data of CfRhPDE1 in comparison with literature values. SD were plotted where available; and *D*, calculated catalytic efficiencies of optogenetic PDEs in overview, along with their expression conditions. CfRhPDE, *Choanoeca flexa* rhodopsin phosphodiesterase; PDE, phosphodiesterase.

## Results

### General structure of CfRhPDE1

The full-length amino acid sequence of CfRhPDE1 (National Center for Biotechnology Information Gene ID: QDH43408.1) was modeled as a dimer using AlphaFold2 (16). This predicted several models, two of which were dismissed (Supporting data) because they were highly implausible: Structure no. 2 located the PDE domain besides the rhodopsin dimer, within the lipid bilayer, making intracellular cGMP hydrolysis (13) impossible. Structure no. 3 was refuted because it shows the rhodopsin domain as a dimer of two rhodopsins with perpendicular orientations of their alpha helices, contradicting decades of rhodopsin research and the crystal structure of the *Salpingoeca rosetta* RhPDE (SrRhPDE) transmembrane domain (17). In contrast, the selected structure (Fig. 1*A*) did comply with established facts about localization and orientation of rhodopsin and PDE domains.

### Biochemical characteristics

We tested all four RhPDE candidate enzymes from *C. flexa* (13) in *Xenopus laevis* oocytes and found that only CfRhPDE1 (Fig. 1*A*) showed significant light-dependent cGMP degradation but no cAMP degradation (Fig. S1, *A* and *E*). In contrast, CfRhPDE2 and CfRhPDE3 did not show any cGMP degradation (Fig. S1, *B*, *C*, *F*, and *G*). Similar findings were observed in HEK293 cells at artificially elevated cAMP levels (18). CfRhPDE4 showed cGMP degradation, but our screening assay did not detect any light dependence (Fig. S1, *D* and *H*). Thus, we focused on CfRhPDE1 and characterized it in ND7/23 cell membranes. Here, we found that the activity changed only slightly in the light at high cGMP (10–100 μM), whereas it proved substantial at lower cGMP concentrations. Variation of the substrate concentrations revealed that the light-dependent fc is realized by means of both the *K*_m_ and *k*_cat_ of the enzyme. Illumination changes the *K*_m_ from 4.4 ± 0.6 μM to 1.9 ± 0.1 μM, whereas the maximum turnover number, *k*_cat_, increases from 2.0 ± 0.1 s^−1^ to 4.7 ± 0.1 s^−1^ (Fig. 1*B*). CfRhPDE1 proved very cGMP-selective, and only very slow cAMP degradation was measurable in the light (*k*_cat_ = 1.1 ± 0.1 s^−1^; *K*_m_ = 505 ± 115 μM) (Figs. 1*B* and S2). Since pyrimidine nucleotides can occur in substantial levels, with cCMP and cUMP even exceeding cGMP in neurons (6), the PDE activity was also tested for these nucleotides, but only negligible hydrolysis rates were found. Specifically, the cUMP hydrolysis at 1500 μM (0.14 nM/s) was almost 6-fold lower than cAMP, and for cUMP, it was again lower at 0.03 nM/s (Figs. S2 and S3). Thus, the CfRhPDE1 catalytic selectivity for cGMP is large with an approximately 500-fold higher preference for cGMP than for cAMP. The catalytic efficiency surpasses LAPD largely and is on par with SrRhPDE (Fig. 1, *C* and *D*). Qualitatively, the dual modulation of CfRhPDE1 is in line with previous observations for other RhPDEs (19) but quantitatively the RhPDEs differ quite substantially (Fig. 1*C* and Discussion). For example, the *K*_m_ values for Sr*RhPDE* are higher, and they decrease from *K*_m =_ 80 μM to 13 μM in the light. The *k*_cat_ = 22 s^−1^ increases to 28 s^−1^ (19).

### Spectroscopic properties

**Figure 2:**
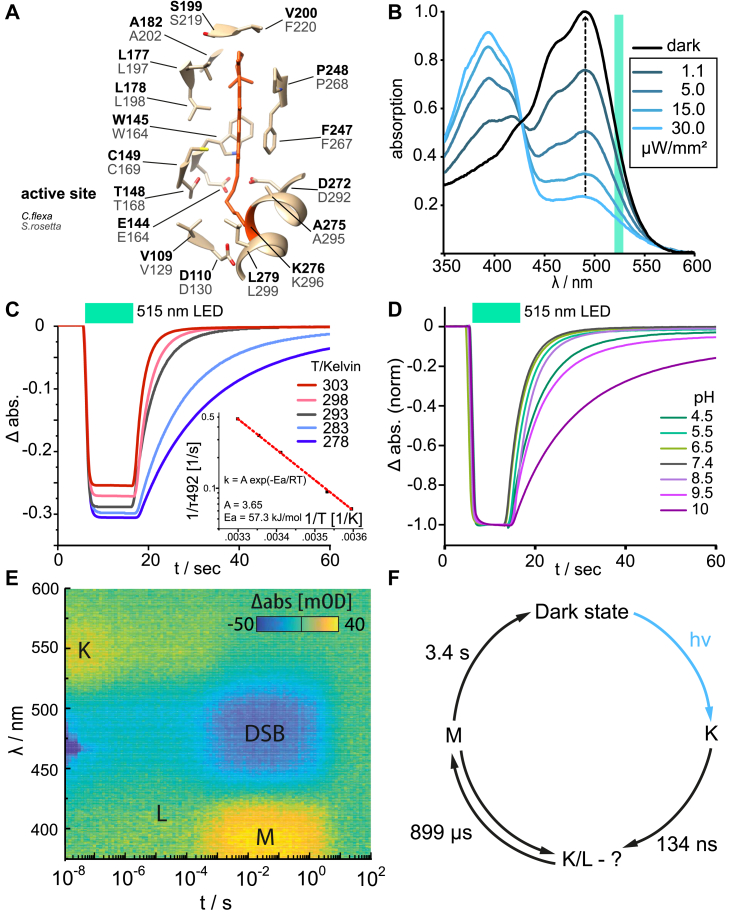
**Structure and spectroscopic properties of CfRhPDE1 transmembrane domain**. *A*, model of the chromophore pocket of the CfRhPDE1 Rh-domain homologous to the SrRh structure (PDB ID:7cj3). Residues in the 5 A° vicinity of the all-*trans* retinal are shown; *B*, steady-state UV-visible absorption spectra of the chromophore region. Dark-adapted state shown in *black*, light-adapted spectra shown in *blue* hues. The sample was illuminated at 530 nm with the light intensities as given in the legend; *C*, temperature-dependent recovery of the 492 nm species; *D*, pH-dependent recovery of the 492 nm species; *E*, time-resolved flash photolysis data; and *F*, photocycle fitted to the data from *E*. PDB, Protein Data Bank.

A homology model for the rhodopsin module CfRh was created based on the SrRh X-ray structure ((17), Protein Data Bank ID: 7cj3) and showed a similar secondary arrangement with eight membrane-spanning alpha helices (Fig. 1*A*). The N terminus of transmembrane helix 0 (TM0) and the C terminus of TM7 are both in the cytosol, and an extracellular helix (ECL0) connects TM0 to TM1. In line with the similar UV-visible spectrum, the residues in the 5 Å vicinity around the chromophore are conserved between *C. flexa* and *S. rosetta* (Fig. 2*A*, (18)). The only exception is V220 at the ß-ionone ring, which is occupied by phenylalanine in *S. rosetta.* We focused on CfRhPDE1 and expressed its transmembrane rhodopsin domain for spectroscopic characterization in *Pichia pastoris*. The purified protein in detergent showed an absorption maximum at 492 nm with a shoulder at 470 and 420 nm (Fig. 2*B*). Constant illumination with 525 nm light gave rise to a main photoproduct with absorption maximum at 390 nm and a shoulder at 420 nm. In the dark, the photoproducts fully relaxed to the original dark state with *τ* = 3.4 s at 20 °C (Figs. 2*C* and S4). The recovery is temperature-dependent with an activation energy of 57.3 kJ/mol at pH 7.4 (Fig. 2*C* inset). The pH dependence is also quite substantial (Figs. 2*D* and S4). To investigate the dynamics of M-state formation, we performed flash photolysis experiments (Fig. 2, *E* and *F*). After excitation, a K-intermediate appeared instantaneously on our time scale of > 1 ns, which absorbs maximally at 550 nm. It converts to an L-type intermediate with *τ* = 125 ns, which subsequently converts again with *τ* = 900 μs to the M-state into a metastable equilibrium. M is the longest-living intermediate and the assumed signaling state reverting to the initial dark state with a time constant of 3.4 s. A similar photocycle and time constant of 4 s for the M-state decay was found in SrRhPDE (20).

### Photomodulation of CNG channels

**Figure 3:**
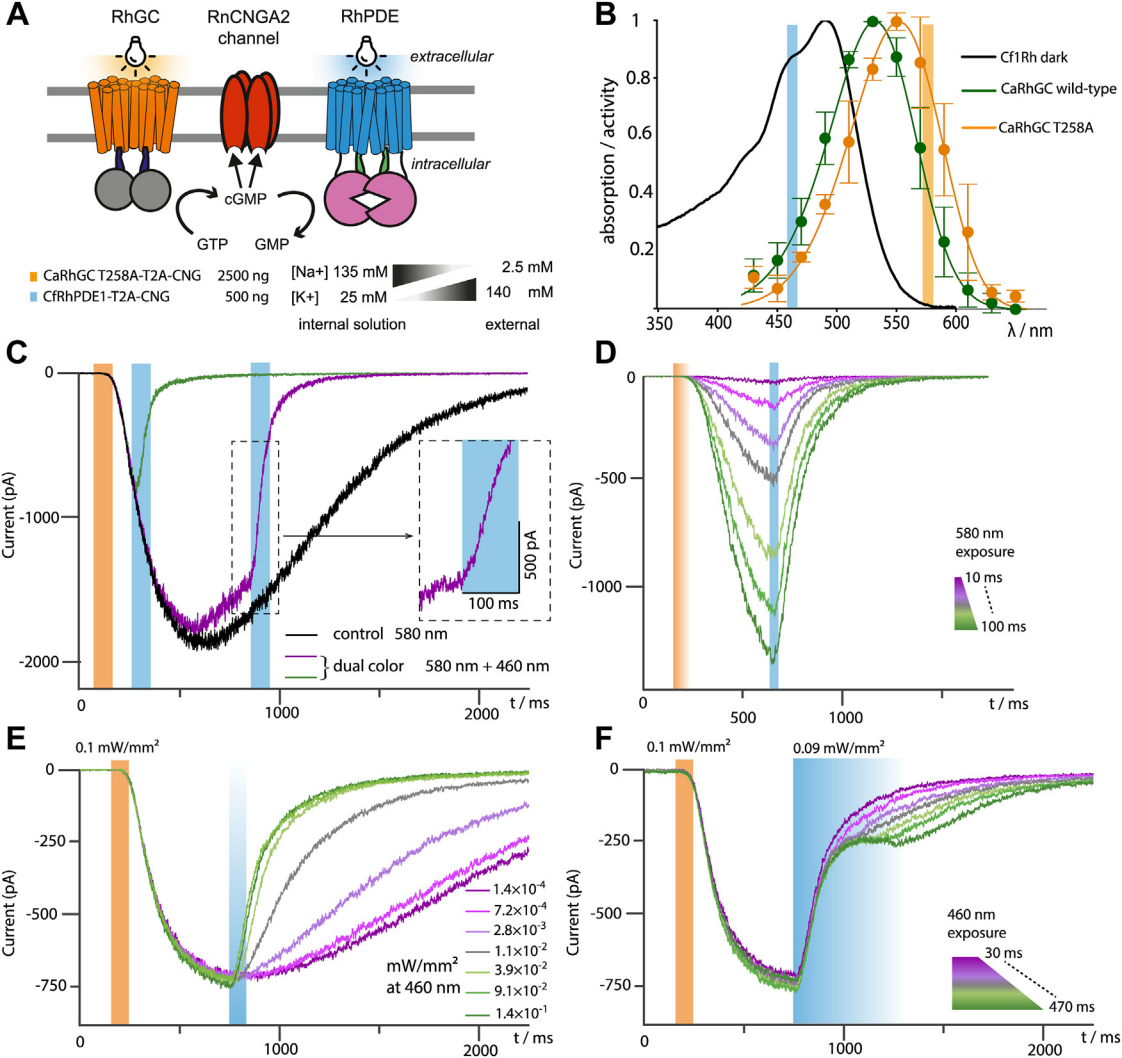
**Bidirectional optogenetic control of cGMP using CfRhPDE1 and CaRhGC to control a CNG channel**. *A*, overview of the expressed recombinant proteins and their respective function, details on transfection conditions and electrophysiology buffers; *B*, comparison of the absorption and action spectra of CfRhPDE1 and CaRhGC (T258A); *C*, default system behavior and dual-color experiment recorded *via* whole-cell two electrode voltage clamp electrophysiology; *D*, influence of the CaRhGC illumination duration; *E*, influence of the CfRhPDE1 illumination intensity; and *F*, influence of the CfRhPDE1 illumination duration. CfRhPDE, *Choanoeca flexa* rhodopsin phosphodiesterase; CNG, cyclic nucleotide-gated ion channel.

Initial biochemical screening and characterization have shown that the enzyme is light-activated with a higher cGMP affinity than SrRhPDE and a *K*_m_ closer to physiological cGMP concentrations in mammals. This result suggested that CfRhPDE1 could be a better choice to control cGMP-regulated effector systems in optogenetic experiments. We chose and coexpressed the Na^+^-selective CNG channel RnCNGA2 (21) from olfactory neurons since the ligand affinity and selectivity are well-known and the inward currents can be precisely monitored by two-electrode voltage clamp measurements (Fig. 3*A*). Furthermore, channel activation and deactivation kinetics are fast, and two-electrode voltage clamp allows for monitoring the rise and decay of cyclic nucleotides with high temporal precision. Considering that the fc of the enzyme is cGMP concentration-dependent *in vitro* (Fig. 1*B*, red line), a high affinity (low *K*_d_) of the downstream CNG channel would also be crucial. Therefore, an electrophysiological experiment was set up using a channel variant with increased nucleotide sensitivity. This was achieved by the channel mutation C460W, known for a 5-10-fold increase in the apparent affinity to cyclic nucleotides (21, 22). As a result, there was improved RhPDE-induced CNG modulation, but small current amplitudes made experiments difficult (data not shown). To address this issue, the channel mutation E342G was added since it removes calcium block effects (22, 23) and currents increased 50-fold to the preferred nA range.

To demonstrate bidirectional and dual-color control of cGMP, we coexpressed the modified RnCNGA2 with CfRhPDE1 and the WT guanylyl cyclase from *Catenaria anguillulae* (CaRhGC) (Fig. 3*A*). However, bidirectional control by different wavelengths was impossible. We reasoned this was caused by a coactivation of both optogenetic enzymes due to their spectral overlap, as the activation spectrum of WT CaRhGC (λ_max_ = 531 nm, Fig. 3*B*) is only slightly red shifted to the CfRhPDE1 absorption (λ_max_ = 492 nm). Thus, at longer wavelengths above ∼570 nm, CaRhGC would be activated almost exclusively, while illumination with wavelengths <510 nm would stimulate CfRhPDE1 primarily. The spectra were further separated to enable dual-color cGMP control by introducing a 23 nm bathochromic shift in CaRhGC by a single mutation (T258A) in the center of helix 3 (Fig. S5). This position is known to induce spectral shifts in other rhodopsins due to a polarity reduction in proximity to the retinal-Schiff base. The corresponding mutation in Chrimson (S169A) caused a comparable shift (24), aligning with this hypothesis. In cells cotransfected with CaRhGC (T258A) and CfRhPDE1 DNA in a ratio of 5:1 (Fig. 3*A*), orange light pulses of 580 nm and 100 ms duration produced large inward currents, which increased for about 500 ms before they slowly declined toward zero within 2 s (Fig. 3*C*, black trace). Due to the co-expression of CfRhPDE1, the application of 460 nm blue light prematurely reduced the cyclase-evoked CNG current (Fig. 3*C*, green and purple traces). The length of the orange activation light pulse (Fig. 3*D*) or intensity or duration of the blue inactivation light (Fig. 3, *E* and *F*) determined the maximal amplitude of the current as well as the on and off kinetics. However, long and intense blue light exposure also activated RhGC due to its residual absorption in the blue range, resulting in intermediate cGMP levels and a corresponding fractional stationary current (Fig. 3*F*). These experiments could be repeated after 10 to 15 s with almost identical characteristics, which implies a reversion of light-adapted CfRhPDE1 to the dark state. Keeping the total DNA amount constant at 3000 ng per cell culture dish (9.2 cm^2^) while transfecting equal amounts of CfRhPDE1 and CaRhGC DNA or even excess CfRhPDE1 DNA could not evoke any inward current upon illumination (see also Discussion). The experiments could also not be performed with *S. rosetta* constructs using the 5:1 DNA transfection scheme (2500 ng of CaRhGC construct and 500 ng of CfRhPDE1 construct). However, with altered amounts of 2000 ng CaRhGC and 1000 ng of SrRhPDE, dual-color photomodulation of the RnCNGA2 channel became possible (Fig. S5 and Discussion).

## Discussion

### Biochemistry of CfRhPDE1

As we have shown above, upon illumination of CfRhPDE1, the *K*_m_ value for cGMP increases about 2-fold, and the maximum turnover number, *k*_cat,_ and *V*_max_ undergo an approximately 2-fold increase. However, CfRhPDE1 in ND7/23 cell membrane fractions reveals much higher affinities than reported previously for this enzyme [5.5 μM in light and 15 μM in darkness, (25)].

The latter values were recorded using *X. laevis* oocyte membrane fractions, and initial experiments in our group pointed to similar values. It seems that the enzyme kinetics of CfRhPDE1 are context-dependent. This is an important warning to be cautious in future investigations of structure–function relationships because it means that kinetic parameters are not absolute and must be considered carefully. They might vary for different expression systems and purification conditions, such as detergent treatment.

The steric changes within the PDE domain of CfRhPDE1 are expected to be similar to SrRhPDE since the latter is also regulated by both *K*_m_ and *k*_cat_ (19). However, the PDE domains differ in the substrate pocket since for CfRhPDE1 the affinity is higher for both the light and the dark state (1.9 and 4.4 μM compared to 13 and 80 μM, respectively). A widely accepted model for the human PDE (HsPDE2A) activation identifies the H-loops in the PDE domains as regulators (3), which brings up a question about a similar regulation of RhPDEs. However, the regulation of the LAPD chimera based on HsPDE2A relies almost entirely on the increase in maximum turnover number *k*_cat_, whereas the *K*_m_ remains almost unchanged (5). Therefore, the RhPDEs from choanoflagellates may form their own class of PDEs.

### Photoswitching of the PDE domain

In a first approximation, the electrophysiological recordings (Fig. 3, *C–F*) monitor cGMP concentration in a time-dependent manner until the saturating concentration of the channel, with the current slope reflecting the net change of cGMP concentration. Since both photoswitching of the PDE and gating of the CNG channel are faster than the observed current change, the observed kinetics in electrical measurements give a lower estimate of the PDE kinetics. Within the first 30 ms of 460 nm illumination, the current slope reaches its maximum (Fig. 3*C* inset), and thus, the conformational changes in the PDE domain must occur faster than 30 ms.

For comparison, we also tested the commercially available and widely used “GloSensor 40F” from Promega in ND7/23 cells. We confirmed the light-induced cGMP formation by CaRhGC (Fig. S7), but the kinetics of the cGMP-induced luminescence after CaRhGC stimulation were slowed down at least 1000-fold compared to our electrophysiology experiments. The cGMP synthesis by chemical stimulation of soluble guanylate cyclases using sodium nitroprusside was also heavily delayed, confirming that the GloSensor is totally inappropriate for kinetic measurements of cGMP production. The GloSensor has a higher *EC*_50_ of 4.5 μM (Promega Inc) compared to the used CNG channel, and therefore, higher cGMP levels are needed to saturate the sensor and overcome its large buffering capacity. However, the high affinity GloSensor variant 42F (*EC*_50_ = 0.18 μM) shows similar slow kinetics, *e.g.*, a signal maximum of 40 min (18) after treatment with 100 μM sodium nitroprusside, which makes buffering effects less plausible. Based on these results, it is almost certain that the slow binding and very slow dissociation rates for the GAF A + B tandem of the GloSensor (26) are the real reason. Additionally, the recombination rate for the split luciferase in the GloSensor (27) slows down the activation kinetics. The discrepancy in kinetics is also apparent with G protein–coupled receptor rhodopsins, where fast kinetics of GIRK channel recordings could not be reproduced using GloSensors (28, 29). Thus, it appears that slow recombination and dissociation rates of the split luciferase are a general limiting factor. The resulting response times are spanning a number of minutes, but the signaling processes investigated should proceed in seconds or subseconds. Consequently, useful luminescent sensors for kinetic studies must rely on fast cyclic nucleotide-binding domains incorporated into a fast split luciferase, as shown recently for a luminescent calcium indicator (30).

Time-resolved infrared spectroscopy could be used to investigate the structural changes in the catalytic site upon cGMP binding, GMP formation, and release on a millisecond time scale. This would require that the RhPDE molecules be synchronized by brief light flashes (31). However, for these experiments, high protein concentrations but low substrate concentrations below the *K*_m_ value are needed, which is an extra hurdle for the analysis of CfRhPDE1 (17). In CfRhPDE1, the excited rhodopsin domain transfers the conformational change to a change in the PDE domain *via* the linker region. The linker contains several fully conserved sections, particularly the cytosolic extension of TM7, ranging from Gln290 to Asn314 and from Leu319 to Lys327 (SrRhPDE numbering, Fig. S8). Given that the substrate affinity is substantially higher than SrRhPDE, but the rhodopsin’s active site (Fig. 2*A*) and spectroscopic characteristics are almost identical, it would be interesting to investigate whether activation of the PDE domain is accomplished by similar mechanisms in these RhPDEs. A full-length structure would be needed, but it still awaits experimental determination.

The reproducibility of the experiments with CfRhPDE1 implies that its PDE domain relaxes within the tested time frame (10–15 s) corresponding to 3 to 4 times *τ* of the M-state (>90% depletion), supporting a direct coupling of the M-state to enzymatic activity (17) and in line with the direct coupling found for CaRhGC (31). From this argumentation, we propose the following model: CfRhPDE1 is only moderately active in darkness. After photoexcitation of the rhodopsin domain, it reaches the signaling M-state with a time constant of 1 ms, which couples directly to the catalytic domain so that a maximum catalytic activity is also reached within 1 ms after illumination. The M-state decays with a time constant of 3.4 s and below 5% within 10 to 15 s, which is equal to the near-basal activity.

### Applicability in optogenetics

The demand for a potent LAPD in optogenetics has not yet been met. However, our electrophysiological data show that rhodopsin-phosphodiesterases enable efficient light sensing and fast cGMP modulation on a subsecond time scale despite a fc of only approximately four, which is lower than previously published LAPDs (5, 19). In general, natural RhPDEs have a high catalytic efficiency, which is about 0.4 to 2.0 μM^−1^ s^−1^ (SrRhPDE, CfRhPDE1, Fig. 1*D*), surpassing the synthetic LAPD (0.14–0.74 × 10^−7^ μM^−1^ s^−1^) approximately 3-fold ((5), Fig. 1*D*). For optogenetics, this is desirable, because it would allow sufficient PDE hydrolysis rates at weak expression levels. It is remarkable that efficient photoswitching can be achieved despite the modest fc of RhPDEs. The concentration level of cGMP is a result of the activities of cyclases and phosphodiesterases, and steady-state is achieved once they are equal in absolute magnitude. Since the concentration of cyclase substrate GTP is kept constant by the patch pipette buffer (refer Experimental procedures section), the cGMP synthesis rate is constant in darkness. With most of the CNG channels closed in the darkness, the cGMP concentration can be assumed to be far below the expected *K*_d_ of the channel, *i.e.*, << 0.3 μM. Stimulation of CaRhGC represents a transient deviation from this artificial equilibrium. Using a high-affinity effector like the presented ion channel, the fc of CfRhPDE1 can be fully exploited to result in a pronounced signal change, as demonstrated by the membrane current modulation. The high affinity of CfRhPDE1 and the operational window of cGMP concentration regimes would also fall within the range of endogenous cGMP levels in neurons, which are submicromolar (32). Additionally, the high selectivity of CfRhPDE1 to cGMP over cAMP, cCMP, and cUMP (Figs. 1*B*, S2, and S3) makes it versatile to probe cGMP effectors in diverse biological systems.

In this setting, the considerable dark activity of CfRhPDE1 is not critical because it further reduces the cGMP dark concentration, whereas high CfRhPDE1 concentrations would substantially reduce the cGMP increase. The L/D fc in YFP-CaRhGC was determined to be 230 in membrane fractions (15). Finding that a five-fold excess of CaRhGC (2500 ng) over CfRhPDE1 (500 ng) was necessary, we suggested that expression ratios are crucial, and that CfRhPDE1 expresses efficiently. This was confirmed by the expression ratio in membranes, which was determined as a 5.3-fold excess of CaRhGC over CfRhPDE1 (Fig. S9). Thus, CaRhGC needs to be expressed in excess over CfRhPDE1 in the optimized experiments. Also, the expression for these two proteins scales similarly with the DNA amount in transfection. This means that they express similarly well, which is a helpful insight for future construct design (refer Outlook). Expression of all of the RhPDEs from *C. flexa* was possible in *X. laevis* cells, as observed by (25). This potentially allows to interchange their rhodopsin or PDE modules to create enhanced chimera. In this sense, CfRhPDE1 and CfRhPDE4 are interesting because both reveal strong cGMP hydrolysis, but only in case of CfRhPDE1, this is light-dependent in host cells (18).

### The role of inactivation kinetics

This three-component system also exemplifies how different off-kinetics affects dual color control over whole cells. CaRhGC is a fast photoreceptor enzyme with a total photocycle duration of 600 ms (33). By the same measure, RhPDE takes 3.5 s (Fig. 2*F*). Due to this, at moderate steady-state 515 nm illumination, when light is absorbed equally well by both enzymes, six times more CfRhPDE1 are active compared to CaRhGC. In line with this, analysis of the current slopes resulting from CfRhPDE1 or CaRhGC activation (Fig. 3, *D* and *E*) reveals a significantly higher operational sensitivity for CfRhPDE1. The *EC*_50_ for CfRhPDE1 is achieved at a photon dose of 2.6 pmol/mm^2^ at 460 nm, whereas it takes 34.6 pmol/mm^2^ at 580 nm for the *EC*_50_ of CaRhGC (Fig. S10) although the relative absorption or activation at these wavelengths are similar [0.9 for CfRhPDE1 and 0.8 for CaRhGC (T258A) (Fig. 3*B*)]. Different off-kinetics not only lead to different steady-state levels, but they also confine the temporal regime for illumination in dual color experiments. Extended illumination at 580 nm excited more red-shifted RhGC in a time-dependent manner (Fig. 3*D*, S10*B*, and S10*C*) and led to larger membrane currents, as expected for a single photoreceptor. In line with this, the CfRhPDE spectrum indicates negligible absorption at long wavelengths (Fig. 3*B*). In contrast, the system responded differently for the second light pulse given at short wavelengths (460 nm excitation). Extended illumination did not lead to increased cGMP degradation (*i.e.*, a more pronounced decrease of the absolute membrane current). In fact, with prolonged 460 nm illumination, the membrane current decreased slower and was even clamped to a new equilibrium level at 250 pA (Fig. 3*F*) due to activation of both enzymes, resulting in a clamped cGMP level. In general, the red-absorbing rhodopsin can be excited exclusively, whereas continuous blue illumination excites both partners in relation to their absorption coefficient at this wavelength and the decay time of the active state.

### Outlook

In contrast to the optogenetic experiment, the original organism expresses all the necessary signaling proteins in stable ratios that are optimal for cellular signaling. Therefore, native photosensing systems can be fine-tuned to work reliably. The three-component system that we present is intricate to set up and could benefit from several improvements. For example, coexpression could be controlled precisely and reliably for the application by carefully selecting promoters and creating stable cell lines. Moreover, they could be expressed as a tandem in a fixed one-to-one ratio, which has been demonstrated recently for channelrhodopsins in BiPOLES (34). The fact that the expression ratio of CaRhGC and CfRhPDE1 (5.3:1) closely follows the DNA ratio (5:1) (Figs. 3*A* and S9) simplifies the design strategies for such bicistronic constructs. However, one to one coexpression will demand that the photocycles of CfRhPDE1 and CaRhGC are equally fast, which would need additional engineering of the constructs (31, 35). An increased fc is also desirable since it would allow a broader variance of expression ratios. To yield such an improved optogenetic PDE, genome mining with subsequent screening and characterization (1) could be beneficial as well, similar to its impact on rhodopsin ion channels and pumps. Also, protein-protein interaction structural studies on the RhPDE dimerization interface of functional CfRhPDE1 may be conducted (36) to unravel whether the regulation mechanism is similar to the proposed “H-loop” regulation in *Hs*PDE2 (4) or not. Alternatively, PDE domains might be designed *de novo* to fit the constraints imposed by photosensory domains such as bacteriophytochromes. Recent methods in the *de novo* design of enzyme domains may be applicable (37). They rely on an established reaction scheme and transition state of the catalyzed reaction, which is available for the cGMP hydrolysis in HsPDE9A (38). Additionally, optogenetics may contribute to the manipulation and investigation of noncanonic cyclic nucleotide signaling (39) since they expand prevailing mass spectrometry techniques (39) by unprecedented spatiotemporal resolution. Furthermore, using optogenetic cCMP/cUMP PDEs and receptors in compartments that have negligible cCMP/cUMP signaling, cells would be endowed with orthogonal optogenetic control. Since this would need the alteration of substrate selectivities of optogenetic PDEs, it would be good to rely on existing knowledge on pyrimidine PDEs. In this line, HsPDE7A may serve as an example for future RhPDE engineering (40).

## Experimental procedures

Reagents were of ACS grade or higher and were purchased from Sigma-Aldrich unless noted otherwise.

### Expression and membrane preparation of full-length RhPDE in ND7/23 cells

CfRhPDE1 was N terminally tagged with Venus fluorescent protein and cloned into the pcDNA3.1 vector without any further targeting sequences and/or alterations to the WT. ND7/23 cells (ECACC 92090903) were grown in Dulbecco’s modified Eagle medium (DMEM, D6429, Sigma-Aldrich) supplemented with 5% fetal bovine serum and 6 μM all-trans retinal to a confluency of 30 to 40%. They were transfected using a 0.1% solution of PEI-40k (#24765, Polysciences) that was prepared according to the manufacturer’s protocol. Per T150 flask, 27 μg of plasmid DNA was mixed with 1500 μl of DMEM. Subsequently, 81 μl of the PEI-40k solution was mixed with 1500 μl DMEM and added dropwise to the DNA solution. The mixture was inverted for mixing, and incubated for 15 min at room temperature. It was spread dropwise over the surface of the cell culture medium. Once they had reached 90% confluency, cells were harvested, and their membranes were extracted similarly to the procedure described (41): osmotic shock, combined with mechanical stress by pushing the volume 4 to 10 times through a 26G syringe sheared and disrupted the cells. Undisrupted cells were removed by centrifugation at 3000 rcf and 4 °C for 10 min. The remaining supernatant was kept and subjected to three rounds of ultracentrifugation at 50,000 rcf and pellet resuspension to purify the membranes. Eventually, the pellet was resuspended, and protein concentration was determined by fluorescence. A commercial phiYFP sample (BioVision) was dissolved in the membrane purification buffer, and its concentration was determined by recording the absorption at 527 nm in an absorption spectrophotometer (UV-2000, Shimadzu). Fluorescence (excitation at 490 nm, 12 nm full width at half maximum) was then recorded for various phiYFP concentrations in a fluorescence spectrophotometer (FluoroMax 4, Horiba), and a calibration slope was fitted. Purified membrane stocks were quantified and diluted accordingly. Finally, CfRhPDE1 was snap-frozen in liquid nitrogen at concentrations between 50 and 500 nM, and aliquots were stored at −80 °C until use.

### Expression and membrane preparation of full-length RhPDE in *X. laevis* oocytes

All four Venus-CfRhPDE constructs were cloned into the pGEM vector for heterologous expression in *X. laevis* oocytes according to the protocols of (33). However, the expression took place in darkness. Cells were selected manually for fluorescence, and membranes were purified following established protocols (19). Absolute CfRhPDE concentrations in the membrane fractions were determined as described for the ND7/23 cells.

### HPLC-based RhPDE *in vitro* assay

For a reaction, 25 μl of purified and diluted CfRhPDE1 membranes were placed in a 96-well plate. For the measurements of light-adapted CfRhPDE1, the enzyme was illuminated by using a LED matrix (part #1487, Adafruit) with the 470 nm channel set to full intensity, resulting in light intensities of 30 μW/mm^2^. Reactions were started by adding 225 μl of reaction buffer with the following composition: 20 mM Hepes, 5 mM DTT, 5 mM MgCl_2_, 100 mM NaCl, and 5% glycerol. The cyclic nucleotides 3′,5′-cAMP, 3′,5′-cGMP, 3′,5′-cCMP (C001, Biolog) and 3′,5′-cUMP (U001, Biolog) were present at defined concentrations ranging from 1.11 to 1665 μM. The final concentrations of CfRhPDE1 ranged from 0.1 to 10 nM and nucleotides from 1 μM to 1500 μM, respectively. The concentration of cyclic nucleotide stocks was measured *via* UV absorption at 259 nm (ε = 15,000 M^−1^ cm^−1^) for cAMP and AMP, 252 nm (ε = 13,500 M^−1^ cm^−1^) for cGMP and GMP, 262 nm (ε = 10,000 M^−1^ cm^−1^) for cUMP and UMP, and 270 nm (ε = 9000 M^−1^ cm^−1^) for cCMP and CMP. Stocks were adjusted to 45 mM (pyrimidine nucleotides) or 100 mM (purine nucleotides). At defined time points between 1 min and 100 min, aliquots of 50 μl were taken and mixed with 9 μl of 1 M HCl to terminate the reaction. Samples were heated for 1 min at 95 °C in a PCR cycler and were pH-adjusted afterward by adding 12 μl of 1 M ammonium phosphate solution (pH 5.0) in case of the purine nucleotide reactions and 12 μl of 1 M potassium phosphate solution (pH 6.8) for the pyrimidine nucleotide reactions. In case of cCMP, 1.2 μl of 1 M triethyl ammonium acetate (TEAAc, 69372, Sigma-Aldrich) stock solution was added. Samples were filtered through a 96-well, 0.22 μm, wwPTFE filter plate (8582, Cytiva) spun at 3000 rcf for 5 min. The ratio of cyclic nucleotides to noncyclic nucleotide monophosphate was determined by HPLC and UV detection in a gradient program. For the purine nucleotides, the mobile phase buffers were as follows: (A) 200 mM ammonium phosphate at pH 5.0 and (B) a 90% acetonitrile/water solution. The gradient method (%A/%B) was as follows: 100/0 from 0 min to 2 min; 100/0 → 92.5/7.5 from 2 min to 3.5 min; 92.5/7.5 from 3.5 min to 5.5 min; 92.5/7.5 → 100/0 from 5.5 min to 7 min; and 100/0 until 17 min. A constant flow rate of 0.238 ml/min was programmed. For the pyrimidine nucleotides, solution A was a 20 mM potassium phosphate/20 mM TEAAc buffer (pH 6.8) and solution B consisted of 90% acetonitrile/10% water/20 mM TEAAc. The corresponding HPLC gradient method (%A/%B) was as follows: 100/0 → 87.5/12.5 from 0 min to 12.5 min; 87.5/12.5 → 100/0 from 12.5 min to 15 min; 100/0 from 15 min to 35 min. The flow rate was 0.5 ml/min. A sample volume of 10 or 20 μl was injected using the HPLC device’s “μl pickup mode”. The HPLC instrument was from Knauer and was equipped with an AS6.1 autosampler, a P6.1L quaternary pump, a 10 mm flow cell, and a column oven. As a stationary phase for the retention of purines, a 150 × 2.0 mm, 2.1 μm ProntoPEARL C18aq narrow-bore column equipped with a 10 x 2.0 mm precolumn was used (Bischoff Analysentechnik). For pyrimidines, a 125 × 3.0 mm, 7 μm Triart Prep C18-S column was used (YMC). The temperature in the column oven was set to 37 °C for purine analysis and 20 °C for pyrimidines. Concentrations were quantified by integration of the respective elution peaks using diode array UV detection. For the purine nucleotides, the detection wavelength was set to 258 nm (8 nm bandwith) with a reference wavelength of 320 nm (20 nm bandwith). For pyrimidine nucleotides, it was set to 270 nm (8 nm bandwith) with a reference wavelength of 340 nm (20 nm bandwith) for pyrimidines. For purines, the method was validated for linear response using GMP, cGMP, AMP, and cAMP standards in a concentration range of 50 nM to 50 μM for 20 μl injection and 50 μM to 1000 μM for 10 μl injection. For pyrimidines, the method was validated for linear response using 3′,5′-cyclic nucleotides and 5′-monophosphate nucleotides in a concentration range of 100 nM to 450 μM for 20 μl injection and 150 nM to 1500 μM for a 10 μl injection. Baseline noise was in the range of 0.01 mAU; limit of detection was defined as 3× baseline noise and limit of quantification as 10× baseline noise. The reaction progress was taken from the ratio of GMP/cGMP and AMP/cAMP, which ruled out the influence of variations in the reaction aliquot volumes or variations in the actual volume injected by the autosampler. The range of < 10% substrate degradation was subjected to a linear fit to determine initial reaction velocities. Three independent reactions were run and analyzed per substrate concentration. These were then averaged, and the standard error was determined. Initial reaction velocities were plotted against initial substrate concentrations and fitted to the Michaelis–Menten equation (7).

The background hydrolysis of cyclic nucleotides caused by endogenous proteins was tested using membranes expressing the catalytically inactive CfRhPDE1 (D575A) mutant. These were prepared and assayed in the same way as described for the WT construct. Background hydrolysis was found to be negligible for purine nucleotides (<1% of WT reaction catalysis). For pyrimidine nucleotides, background hydrolysis ranged from 0.09 to 0.54 nM/s at 0.25 mg total membrane protein per ml and was taken into account accordingly for the WT reactions.

### ELISA-based RhPDE *in vitro* screening assay

The screening assay was identical to the HPLC-based *in vitro* assay in the following ways: illumination device and multiwell plate, source and preparation of cGMP stocks, and final concentrations of CfRhPDE proteins in the reaction pot. For each reaction, 10 μl of the membrane fractions of CfRhPDE proteins with concentrations of 80 nM RhPDE were placed in each well, and 190 μl of reaction buffer were added to start the reaction. At indicated time points, aliquots of 10 μl were drawn and mixed with 500 μl of 0.1 M HCl to terminate the reaction. In addition, 9.5 μl of the pure reaction buffer was mixed with 0.1 M HCl to measure the initial cGMP concentration. Absolute cGMP levels in the aliquot samples were determined using a commercial ELISA kit (K020-C1, Arbor Assays). To optimize accuracy, samples were diluted to concentrations within the steepest range of the dose-response curve of this assay, which spans 1 nM to 100 pM in the nonacetylated format used. Stock samples for calibration spanned a range of 150 PM to 2 nM, and samples were measured in duplicate.

### Expression and purification of the transmembrane domain

Briefly, the RhPDE transmembrane domain was N terminally tagged with a 6xHistidine tag and cloned into the pPICZ vector for expression in the yeast *P. pastoris* (clone SMD1168H, Thermo Fisher Scientific), solubilized in a mixture of n-Dodecyl ß-maltoside (DDM, D97002-C, Glycon Biochemicals GmbH) and cholesteryl hemisuccinate. It was then affinity-purified on a nickel column following established protocols (42).

### UV-visible absorption spectroscopy

Absorption spectra of purified transmembrane domain were obtained in a buffer containing 50 mM Hepes (pH 7.4), 200 mM NaCl, and 0.02% DDM/0.004% cholesteryl hemisuccinate using a Shimadzu UV-2000 photospectrometer with UVProbe v2.34 also from Shimadzu Corporation. For the recording of light-adapted UV-visible spectra, the cuvette was illuminated at different intensities of a KL 1500 Light Source (Schott) with a 525/15 nm bandpass filter inserted. The light intensities at the level of the cuvette volume were quantified using a P-9710 optometer (Gigahertz-Optik). Flash photolysis experiments were performed as described previously (43).

### Determination of expression levels and ratios

To determine the expression ratios of CaRhGC_T258A and CfRhPDE1, fluorescence spectrometry was used. ND7/23 cells were grown in a T150 cell culture flask (TPP) until 70% confluency and were then transfected with the Venus-CfRhPDE1 and mTurquoise2-CaRhGC constructs from the electrophysiology experiments. Briefly, 7500 ng of CfRhPDE1 plasmid and 37,500 ng of CaRhGC plasmid were transfected using 90 μl of FuGENE HD and 1500 μl of autoclaved milliQ water. In accordance with the electrophysiology experiments, cells were grown for 45 to 55 h to allow expression and were then harvested. In addition, the mTurquoise2-CaRhGC construct alone was transfected and expressed in a T25 flask (TPP) using the PEI protocol from the *in vitro* experiments. Cell membranes were purified as described before. Using a fluorescence spectrophotometer (FluoroMax 4, HORIBA), emission spectra of mTurquoise2 and Venus alone was recorded using the mTurquoise2-CaRhGC expression and the Venus-CfRhPDE1 membranes from the *in vitro* experiments. The spectra were first scaled according to their molecular brightness to calculate various linear combinations and then normalized to their isosbestic point at 514 nm. A fluorescence spectrum of the membranes with the coexpressed construct was recorded using excitation at 472 nm (where relative excitabilities for the two fluorophores are equal) and scaled to the isosbestic point. The input and exit slit width was 2 nm for all spectra. For each spectrum, six spectra were averaged, and the average of six blank spectra was subtracted. The best fit for a linear combination matching the coexpression spectrum was found manually with interval nesting to a precision of 0.1 units in expression ratio. The total level of membrane proteins was quantified using a commercial protein assay (Thermo Fisher Scientific 23225) according to the manufacturer’s protocol.

### Molecular biology and cloning

The sequences encoding for CfRhPDE1, CaRhGC, and RnCNGA2 were ordered codon-optimized to *Homo sapiens* and cloned into the pcDNA3.1 vector (*in vitro* assay), pEGFP vector (electrophysiology in ND7/23 cells), or pPICZ vector (Rh module purification from *P. pastoris*). The sequences of Venus and mTurquoise2 fluorescent proteins were joined *via* a *XhoI* linker (peptide sequence: LE) to the Rhodopsin enzyme sequences, which were finally joined *via* a GSG/T2A linker (peptide sequence: ELGGGSGGGEGRGSLLTCGDVEENPGPRT) to the RnCNGA2 sequence.

### Electrophysiology in ND7/23 cells

Electrophysiological traces were recorded using whole-cell patch-clamp electrophysiology in whole-cell voltage clamp mode. Patch pipettes with a resistance of 2 to 3.5 MΩ were prepared from borosilicate capillaries (GB150P-9P, Science Products GmbH), using the P-1000 Micropipette Puller (Sutter Instrument Co). ND7/23 cells (ECACC: 92090903; Sigma-Aldrich) were seeded 72 h before experiment on coverslips placed in 34 mm plastic cell culture dishes (TPP) filled with 2 to 2.5 ml of culture medium. Subsequently, 45 to 55 h before experiments, they were transfected with the indicated plasmids in absolute ratios of 2500 ng: 500 ng (CaRhGC-T2A-RnCNGA2 (E342G, C460W): CfRhPDE1-T2A-RnCNGA2 (E342G, C460W)) or 2000 ng: 1000 ng (CaRhGC-T2A-RnCNGA2 (E342G, C460W): SrRhPDE-T2A-RnCNGA2 (E342G, C460W)) using FuGENE HD Transfection Reagent (Promega), 6 μl reagent: 250 μl autoclaved milliQ water, with the general procedure according to the manufacturer’s protocol.

Recordings were taken at room temperature using a MultiClamp 200B Amplifier and digitized with the Digidata 1550B digitizer (both from Molecular Devices). The cells recorded had a minimum membrane resistance of 400 MΩ and an access resistance below 10 M Ω. Data were acquired at 10 kHz and filtered at 1 kHz. After a successful patch, cells were given 1 to 2 min for proper exchange of the capillary’s intracellular buffer with the cytoplasm before the first recording was taken. The minimum waiting time between sweeps and recordings was 15 s to allow for full recovery of CfRhPDE1. Buffers were composed as follows: intracellular buffer: KGluc 135 mM, MgCl_2_ 4.6 mM, Hepes 17.8 mM, EGTA 1 mM, MgATP 4 mM, NaGTP 0.3 mM, phosphocreatine disodium salt 12 mM, and phosphocreatine kinase 50 U/ml, pH 7.3, 300 mOsm; and extracellular buffer: NaCl 140 mM, KCl 2.4 mM, MgCl_2_ 4 mM, CaCl_2_ 2 mM, glucose 10 mM, and Hepes 10 mM, pH 7.4, 300 mOsm. Buffers were filtered through 0.2 μm pore filters and stored at −20 °C until use.

Illumination periods were controlled by a VS25/VCM-D1 shutter system (Vincent Associates), and light was delivered through an inverse microscope as epi-illumination. The optical setups used were as follows: for Figure 3*C*: fluorescence excitation light and actinic light were delivered by a monochromator (OptoScan, Cairn Research Ltd), and an IX70 inverted microscope (Olympus) was equipped with a 40× immersion objective (UApo/340 40×/1.15W, Olympus); and for Figure 3, *D–F*: a Polychrome V monochromator (TILL Photonics) was used in combination with an AxioVert 100 inverted microscope (Carl Zeiss) equipped with a 40× immersion objective (W Plan-Apochromat 40×/1.0, Carl Zeiss). Light intensities were regulated by a twisted nematic liquid crystal cell (Adafruit) inserted into the beam path. We applied an analog control voltage between 0 and 3 V during shutter opening to change its transmittance. For both setups, absolute light intensities were measured on top of a coverslip with the immersed objective beneath using a P9710 optometer (Gigahertz-Optik) and normalized to the respective objective illuminated field.

## Data availability

The data are deposited in this manuscript at JBC.

## Supporting information

This article contains supporting information (18, 25).

## Conflict of interest

The authors declare that they have no conflicts of interest with the contents of this article.

## Supporting information

RhPDE_SUPP_rev1_v9.0-newVersion

SUPP_p1_v9.0_rev1_pfade

SUPP_p2_v9.0_rev1_pfade

SUPP_p3_v9.0_rev1_pfade

SUPP_p4_v9.0_rev1_pfade
