# Supplementary material for: Characterization of a rhodopsin-phosphodiesterase from Choanoeca flexa to be combined with rhodopsin-cyclases for bidirectional optogenetic cGMP control
Source: J Biol Chem. 2025 Mar 11;301(4):108401. doi: 10.1016/j.jbc.2025.108401 (PMC12004702; doi:10.1016/j.jbc.2025.108401)
Supplement: RhPDE_SUPP_rev1_v9.0-newVersion [file mmc1.docx]

**Supporting information**

**S1, ELISA-based in vitro screening data on four CfRhPDE proteins.** CfRhPDE proteins in membrane fractions purified from *X.laevis* oocytes, final CfRhPDE concentrations in reaction pot: 8 nM each. Reactions were sampled at the indicated time points and measured in duplicates. *A,E:* Observed time course for CfRhPDE1 at 100 µM and 10 µM initial cGMP. Note that cGMP was below the limit of detection for t = 180 s and 360 s in *E.* *B, F*: Data for CfRhPDE2; *C, G*: Data for CfRhPDE3; *D, H*: Data for CfRhPDE4. Note that for CfRhPDE4, cGMP hydrolysis was detected, but light-independent. Numbering identical to (18, 25). NCBI Gene ID: QDH43408.1 for CfRhPDE1, QDH43410.1 for CfRhPDE2, QDH43407.1 for CfRhPDE3, and QDH43409.1 for CfRhPDE4.

**S2, Enzyme kinetic data of HPLC-based cAMP in vitro assay and selected cUMP activities.** Data were normalized to 1 nM enzyme concentration. n=1 for each nucleotide reaction tested; linear slope fit as described in the Experimental procedures.

**S3, Enzyme kinetic data of HPLC-based in vitro assay for cCMP and cUMP.** Data were normalized to 1 nM enzyme concentration of CfRhPDE1. n=1 reactions for each nucleotide reaction; linear slope fit as described in the Experimental procedures. Plotted is the error of the linear fit. n.d.: no hydrolysis product detectable.

**S4, Spectral recovery kinetics of the light-adapted state of Cf1Rh at different pH.** Recording wavelength: 492 nm, biexponential fit at different pH

**S5, Retinal binding pocket of CaRhGC wt with T258**. Indicated are the residues in the vicinity of the retinal chromophore, T258 is located at the retinal schiff base and was mutated to Alanine.

**S6, Proof of *S.rosetta* RhPDE applicability in electrophysiology.** Dual-color experiment with CaRhGC(T258A), SrRhPDE1 and RnCNGA2(E342G,C460W). Transfection conditions as described in the Experimental procedures.

**S7, Initial experiments reveal slow on-kinetics of ‘glo-sensor’ 40F.** ND7/23 cells were seeded in a 96-well plate and transfected with different constructs. Cells transfected with CaRhGC were kept dark or stimulated with 520 nm light for 2 min and compared to SNP-stimulated cells as a reference.

**S8, sequence alignments of CfRhPDE1 and SrRhPDE1.** Structural features assigned based on crystal structures for *S.rosetta* Rh module and PDE domain (PDB ID: 7cj3, 5vyd), and AlphaFold 2.0 model of CfRhPDE1. Coloring scheme as in Fig.1*A*: *Black*, N-terminus; *Blue*, Rh module; *Green*, linker region; *Purple*, PDE domain; and *Orange*, Retinal-binding Lysine.

**S9, Determination of relative expression levels of mTurquoise2-CaRhGC(T258A) and Venus-CfRhPDE1** *Red and blue solid lines*: relative fluorescence spectra of mTurquoise2 and Venus were scaled according to their molecular brightness, and then normalized to their intersection at 514 nm. *Dashed lines:* Linear combinations of those spectra were normalized to the isosbestic point at 514 nm and illustrate example expression ratios. *Black solid line*: Fluorescence spectrum of the actual coexpression normalized to the isosbestic point at 514 nm. For methodical details, refer to the main text.

**S10, Light titration for CfRhPDE1 and CaRhGC from electrophysiology.** *A*, *Cf*RhPDE1 stimulation by 460 nm light with various intensities. The current traces in the boxed region were subjected to a linear slope fit. (zoom-in of Fig. 3*E*); *B*, CaRhGC stimulation by 580 nm light with various durations. The current traces in the boxed region were subjected to a linear slope fit. (zoom-in of Fig. 3*D*); *C*, dose-response plot of the retrieved current slopes over the applied photon doses. Data were fitted to a Hill function.
