# Supplementary figures and images for: Characterization of a rhodopsin-phosphodiesterase from Choanoeca flexa to be combined with rhodopsin-cyclases for bidirectional optogenetic cGMP control
Source: J Biol Chem. 2025 Mar 11;301(4):108401. doi: 10.1016/j.jbc.2025.108401 (PMC12004702; doi:10.1016/j.jbc.2025.108401)

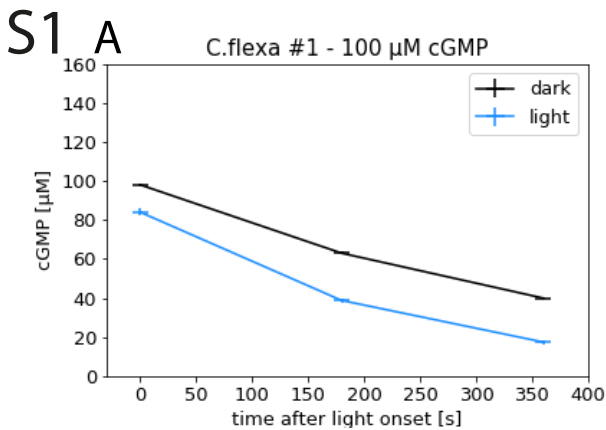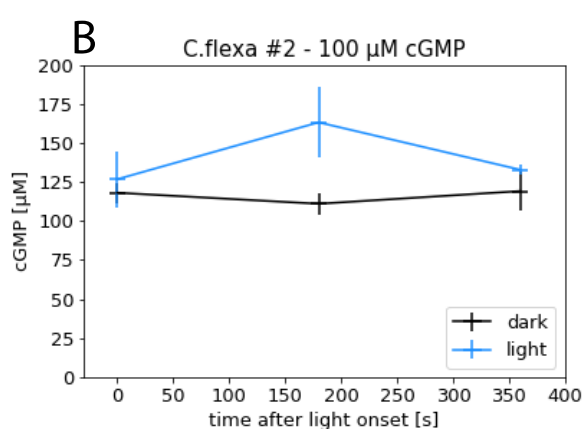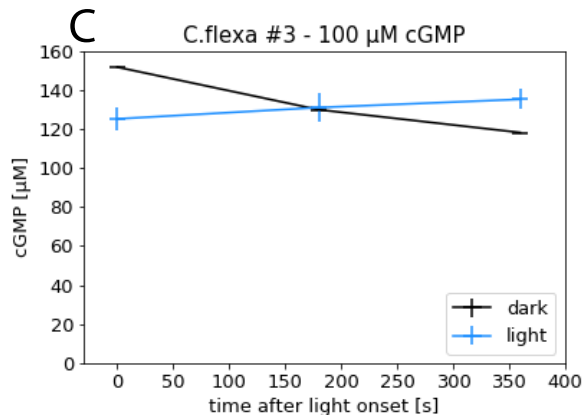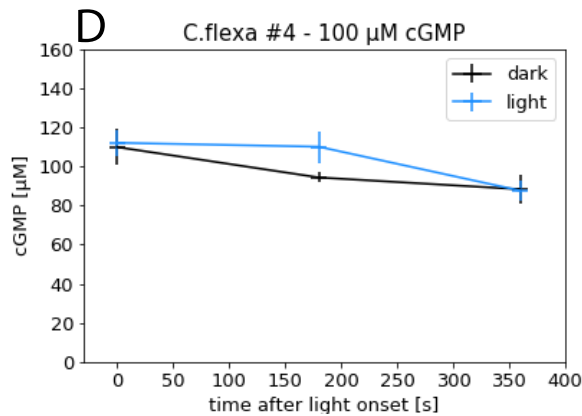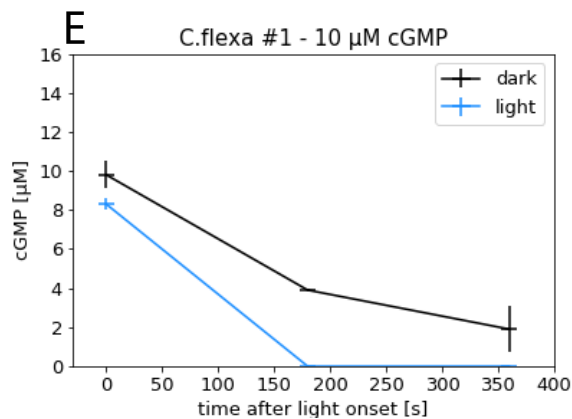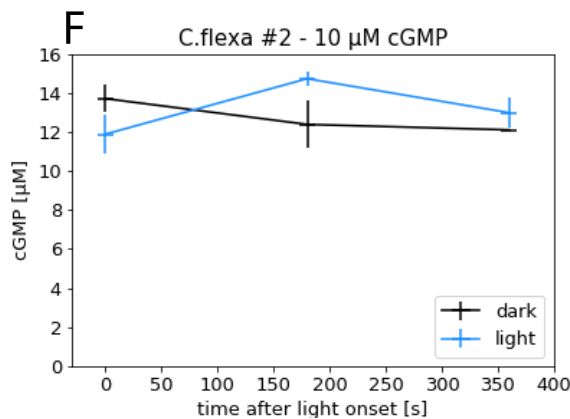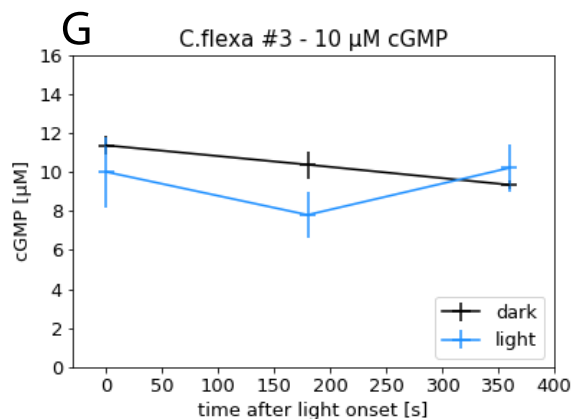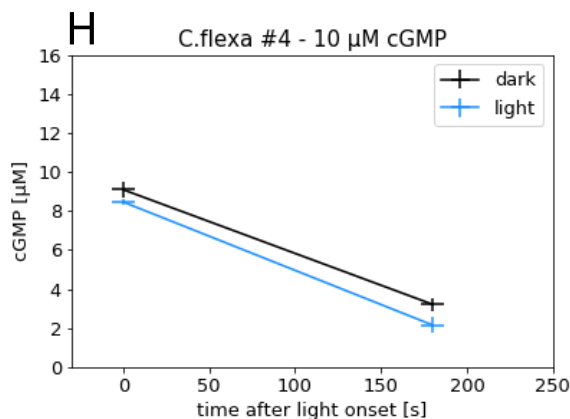

Supplement: SUPP_p1_v9.0_rev1_pfade [file mmc2.pdf]

S2

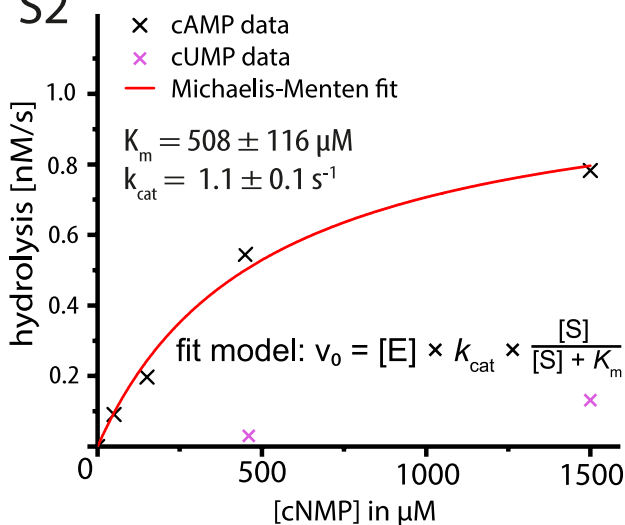

S3

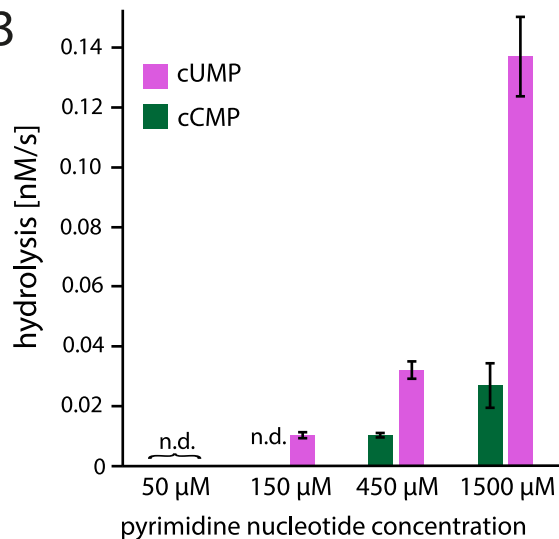

S4

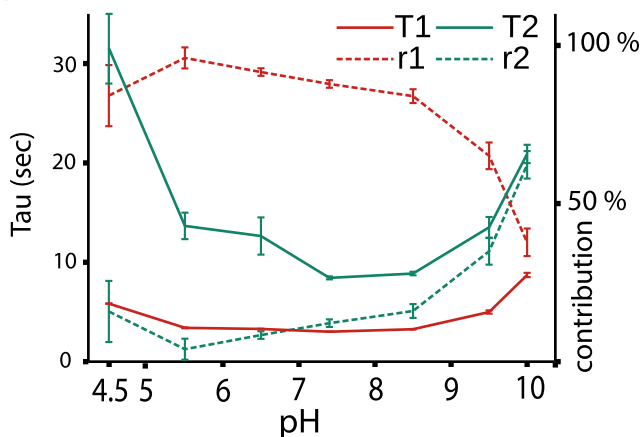

S5

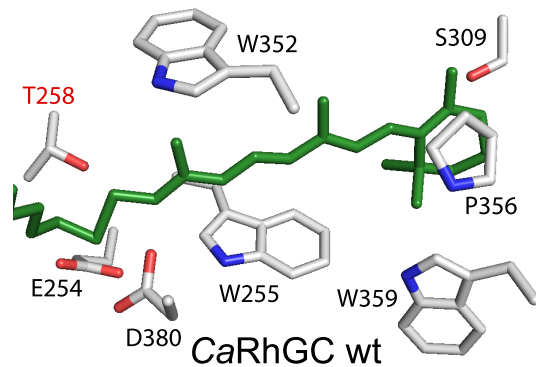

S6

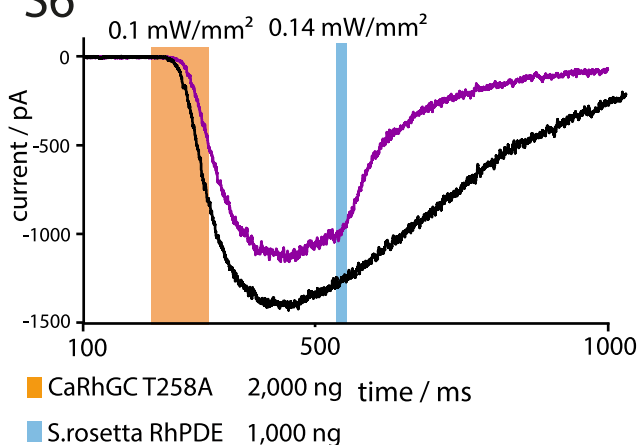

S7

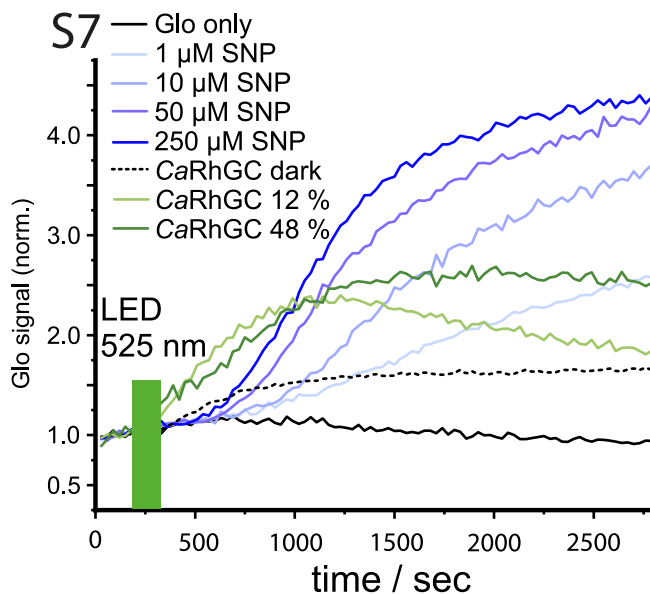

Supplement: SUPP_p2_v9.0_rev1_pfade [file mmc3.pdf]

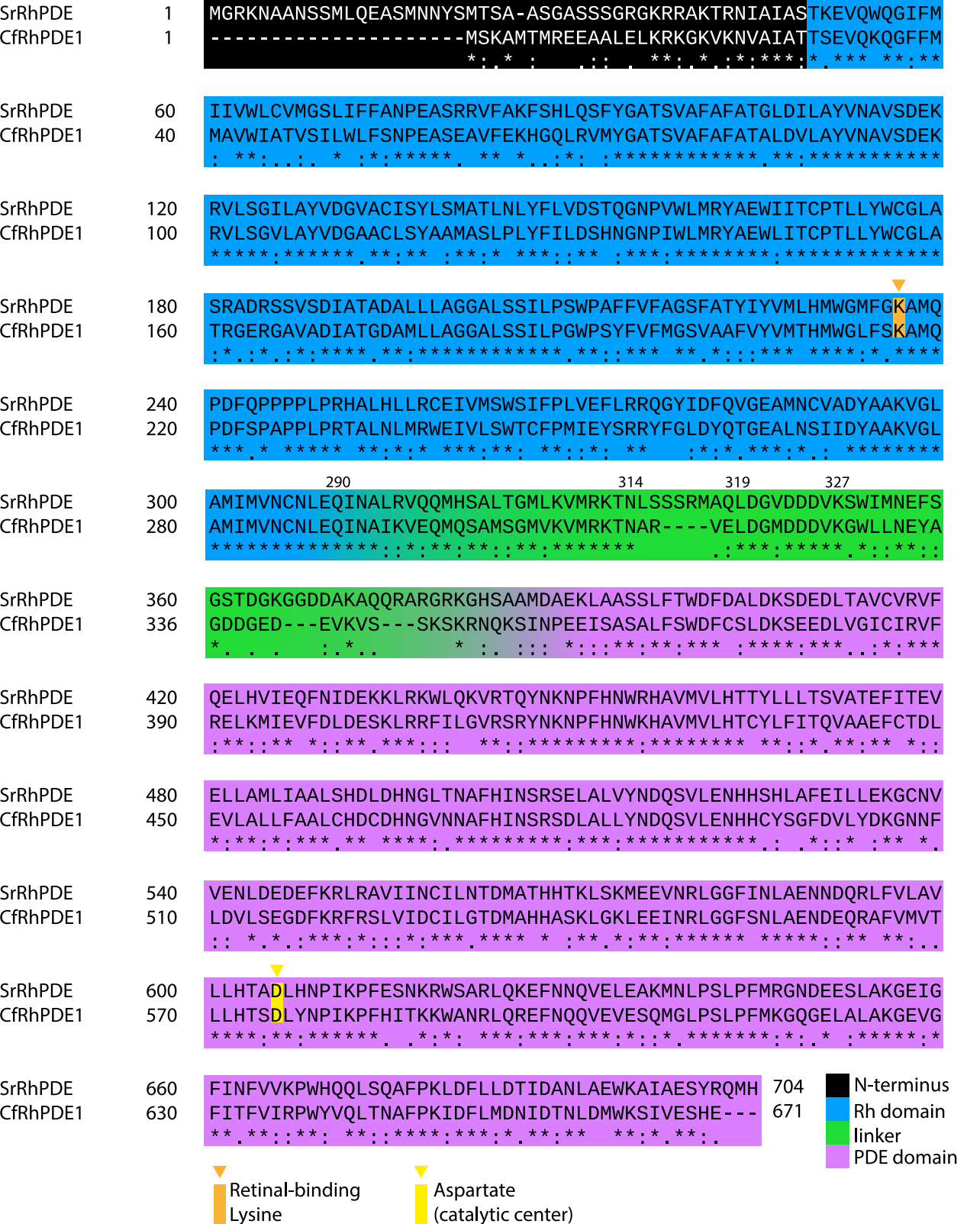

Supplement: SUPP_p3_v9.0_rev1_pfade [file mmc4.pdf]

S9

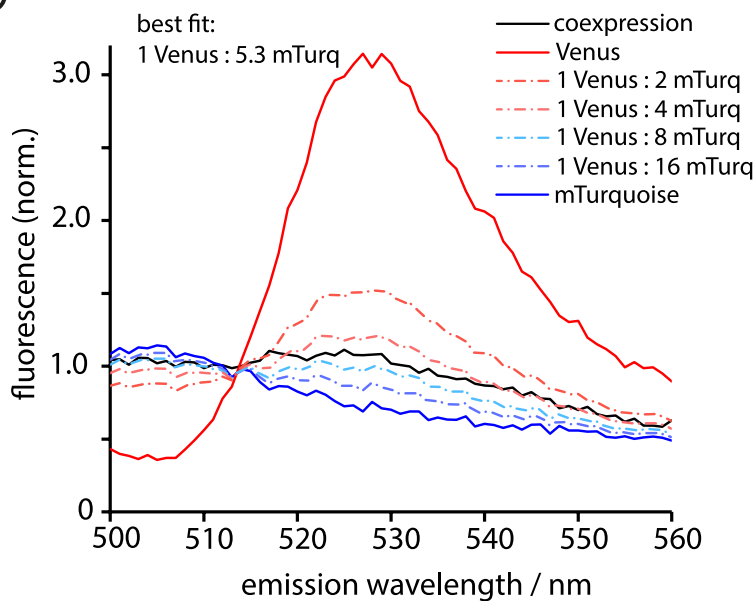

S10

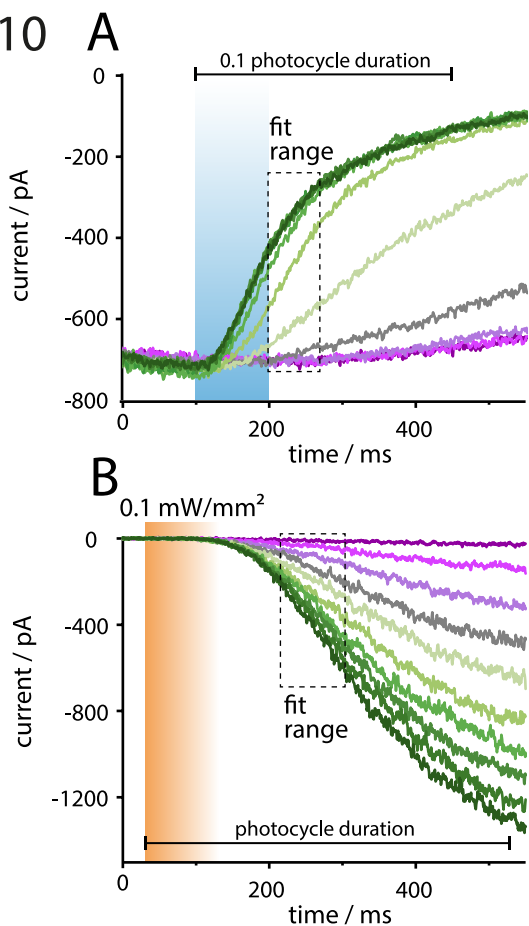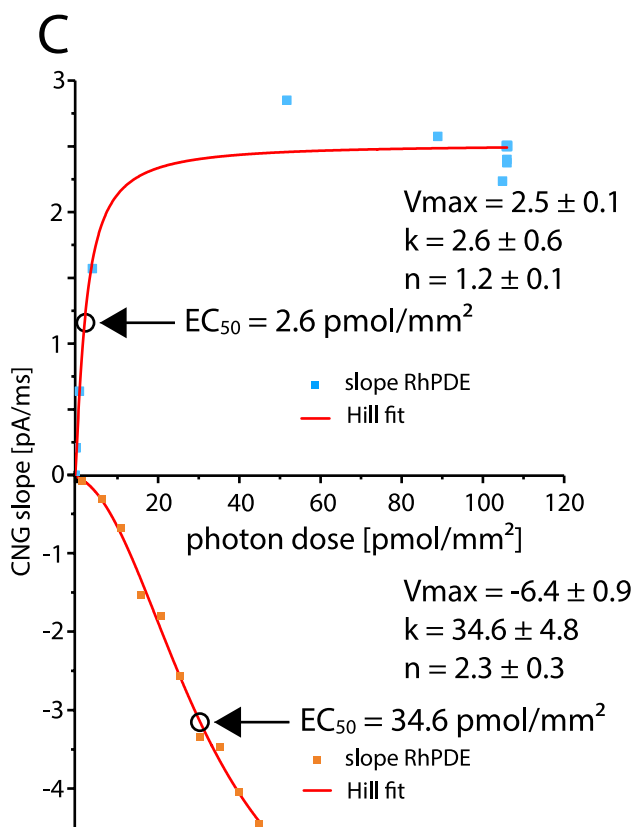

Supplement: SUPP_p4_v9.0_rev1_pfade [file mmc5.pdf]
